# High Hepatitis E Virus (HEV) Seroprevalence and No Evidence of HEV Viraemia in Vietnamese Blood Donors

**DOI:** 10.3390/v15102075

**Published:** 2023-10-11

**Authors:** Le Chi Cao, Vanessa Martin, Le Thi Kieu Linh, Tran Thi Giang, Ngo Thi Minh Chau, Ton Nu Phuong Anh, Vu Xuan Nghia, Nguyen Trong The, Truong Nhat My, Bui Tien Sy, Nguyen Linh Toan, Le Huu Song, C.-Thomas Bock, Thirumalaisamy P. Velavan

**Affiliations:** 1 Institute of Tropical Medicine, University of Tübingen, 72074 Tübingen, Germany; lechicao@hueuni.edu.vn (L.C.C.); vanessa.martin@student.uni-tuebingen.de (V.M.); lk.linh1509@gmail.com (L.T.K.L.); 2 Department of Parasitology, Hue University of Medicine and Pharmacy (HUMP), Hue University, Hue 49000, Vietnam; ttgiang.med@hueuni.edu.vn (T.T.G.); ntmchau@hueuni.edu.vn (N.T.M.C.); tnphuonganh@hueuni.edu.vn (T.N.P.A.); 3 Vietnamese-German Center for Medical Research (VG-CARE), Hanoi 10000, Vietnam; drthe108@gmail.com (N.T.T.); truongnhatmy@gmail.com (T.N.M.); tiensy2015@yahoo.com (B.T.S.); lehuusong@108-icid.com (L.H.S.); 4 108 Military Central Hospital, Hanoi 10000, Vietnam; nghia69@gmail.com; 5 Department of Pathophysiology, Vietnam Military Medical University, Hanoi 10000, Vietnam; toannl@vmmu.edu.vn; 6 Division of Viral Gastroenteritis and Hepatitis Pathogens and Enteroviruses, Department of Infectious Diseases, Robert Koch Institute, 13353 Berlin, Germany; 7 Faculty of Medicine, Duy Tan University, Danang 550000, Vietnam

**Keywords:** hepatitis E virus, blood donors, transfusion, seroprevalence

## Abstract

The prevalence of hepatitis E virus (HEV) in the Vietnamese population remains underestimated. The aim of the present study was to investigate the seroprevalence of HEV IgG/IgM antibodies and the presence of HEV RNA in blood donors as a part of epidemiological surveillance for transfusion-transmitted viruses. Serum samples from blood donors (*n* = 553) were analysed for markers of past (anti-HEV IgG) and recent/ongoing (anti-HEV IgM) HEV infections. In addition, all serum samples were subsequently tested for HEV RNA positivity. The overall prevalence of anti-HEV IgG was 26.8% (*n* = 148/553), while the seroprevalence of anti-HEV IgM was 0.5% (*n* = 3/553). Anti-HEV IgG seroprevalence in male and female donors was similar (27.1% and 25.5%, respectively). A higher risk of hepatitis E exposure was observed with increasing age. None of the blood donors were HEV RNA positive, and there was no evidence of HEV viraemia. Although the absence of HEV viraemia in blood donors from Northern Vietnam is encouraging, further epidemiological surveillance in other geographical regions is warranted to rule out transfusion-transmitted HEV.

## 1. Introduction

Hepatitis E virus (HEV) is a major cause of acute viral hepatitis and is mainly transmitted through contaminated water or food, especially in areas with poor sanitation. HEV infections affect about 20 million people in developing countries each year, with more than 3 million symptomatic cases and 44,000 reported deaths [1]. In most cases, hepatitis E is a self-limiting disease. However, in pregnant women, organ transplant recipients, people with underlying liver disease, and HIV-infected and immunocompromised patients, the disease can be more severe [2]. HEV is an enterically transmitted small quasi-enveloped or non-enveloped single-stranded RNA virus and belongs to the Hepeviridae family with several species in the subfamily *Orthohepevirinae*. Currently, five genotypes (HEV-1 to HEV-4, and HEV-7) classified in the genus *Paslahepevirus* are known to infect humans [3]. HEV genotype 1 and genotype 2 (common in Asia and Africa) are mainly transmitted via the faecal–oral route and lead to acute hepatitis [2,4]. HEV genotype 3 and genotype 4 (in developed and developing countries) are transmitted mainly through the consumption of contaminated food, especially raw or undercooked pork. Additionally, other animal products such as liver pâté, sausage, or fresh shellfish are often associated with sporadic human cases [2,5]. Infection with HEV-7 occurs through the consumption of HEV-contaminated camel products, which are common in some regions of the Middle East [2]. In Vietnam, only HEV genotypes 3 and 4 are currently circulating, suggesting predominantly zoonotic transmission of HEV in this country [6,7]. Testing of blood and stool samples is valuable for understanding transmission patterns and monitoring the prevalence of HEV. HEV can be detected in both blood (at 2 to 6 weeks) and stool (in the acute phase) during the course of the infection [2]. The virus occurs as quasi-enveloped virus particles in blood and urine and as non-enveloped virions in bile and faeces [8]. Blood transfusions can be a potential route of transmission, and performing HEV screening on blood donations, especially in regions where HEV is endemic, can minimize the risk of transfusion-transmitted infection [9]. A first report involved a patient in Japan, who developed acute hepatitis E after transfusion of HEV-positive blood products in 2004 [10]. Since that time, studies have accumulated showing an increasing incidence of transfusion-transmitted HEV and associated risks in immunocompromised individuals [11]. Certain individuals, including pregnant women, immunocompromised individuals, and patients with pre-existing liver disease, may be more vulnerable to severe complications if they receive blood from an infected donor.

Many European countries have introduced HEV testing for blood donations [2]. More than 3.2 million blood donor samples have been tested for HEV RNA in the European Union, and one study reports that 1 in 3109 donations is HEV RNA positive [12]. As more cases of transfusion-transmitted HEV are reported in Asia [13,14,15], transfusion safety is a challenge in countries with dynamic health policies and practices. Vietnam is an HEV endemic area in Southeast Asia with high rates of faecal–oral and animal transmission [7], making hepatitis E a significant public health problem in a country with high HBV prevalence [16]. A previous study has shown that HEV superinfection can worsen the clinical course and progression of HBV-related liver disease in a population [16]. As part of an epidemiological surveillance for transfusion-transmitted viruses, the study aimed to investigate the prevalence of HEV IgG/IgM antibodies and the presence of HEV RNA in blood donors.

## 2. Materials and Methods

### 2.1. Ethics Statement

The study was approved by the ethics committee of 108 Military Central Hospital, Hanoi, Vietnam (108/RES/OBI-HEP-VGCARE-V-D1-20-09-2021). Informed written consent was obtained from all donors. All experiments were performed following GCP/GCLP guidelines.

### 2.2. Study Cohort

The blood donation for this cross-sectional study was obtained from the Hematology and Blood Transfusion department of the 108 Military Central Hospital, Hanoi, Vietnam, in 2021. Based on the studies conducted in the region [16] and the sample size calculation, we hypothesized that we needed to screen blood donors (*n* = 553) (with a power of 80% and a two-sided confidence interval of 95%) to determine the seroprevalences. Demographic data and blood samples (*n* = 553) were collected from healthy adult donors mostly belonging to the Kinh ethnicity. In accordance with standard hospital algorithm, all blood donor samples were serologically tested for HIV, HCV, and HBV (HBsAg) and were confirmed to be negative (using VITROS Immunodiagnostic Products HBsAg, Anti-HIV 1 + 2, and Anti-HCV from Ortho-Clinical Diagnostic, Felindre Meadows, Bridgend, UK).

### 2.3. Serological Assays

The blood donor serum samples were screened for anti-HEV IgG and anti-HEV IgM antibodies using specific enzyme-linked immunosorbent assay (ELISA) (WANTAI, Beijing, China). The Wantai HEV test was selected for our analysis based on our own interlaboratory tests and on the sensitivity and specificity performances of the assays used in many studies in Europe and Asia. The anti-HEV IgG (sensitivity of 99.08% and specificity of 99.9%) and anti-HEV IgM (sensitivity of 97.1% and specificity of 98.4%) ELISA assays were previously evaluated in multicentre clinical trials involving acute hepatitis E samples, samples from individuals living in hepatitis E outbreak areas, and 10,587 blood donation samples (also refer the following: https://www.ystwt.cn/wp-content/uploads/2018/04/Wantai-HEV-IgG-ELISA.pdf; accessed on 3 July 2023). The ELISA protocol was followed according to the manufacturer’s instructions. The test was considered positive if the optical density was ≥0.4 + non-reactive control (NRCx) and ≥0.5 + NRCx for IgM and IgG, respectively. Absorbance was measured with a CLARIOstar microplate reader (BMG Labtech, Ortenberg, Germany).

### 2.4. Nucleic Acid Isolation

Nucleic acid isolation from serum samples was conducted using QIAmp Viral RNA Mini Kit (Qiagen GmbH, Hilden, Germany) according to the manufacturer’s protocol. For the isolation, 140 µL of serum was used, and the nucleic acids were eluted in 60 µL of elution buffer. The quality and quantity of the RNA were measured using NanoDrop™ (Thermo Fisher Scientific, Waltham, MA, USA) and stored at −80 °C until use.

### 2.5. HEV-Specific RT-PCR

HEV RNA was reverse transcribed into cDNA using a High-Capacity cDNA Reverse Transcription Kit (ThermoFisher Scientific, Waltham, MA, USA). The presence of HEV RNA was examined using a nested polymerase chain reaction (PCR) assay using primers targeting the conserved regions of the overlapping HEV ORF1 as described in Hoan et al. [16]. The outer primer pairs were HEV-38 (sense) 5′-GAGGCYATGGTSGAGAARG-3′ and HEV-39 (antisense) 5′-GCCATGTTCCAGACRGTRTTCC-3′; while the inner primers were named as HEV-37 (sense) 5′-GGTTCCGYGCTATTGARAARG-3′ and HEV-27 (antisense) 5′-TCRCCAGAGTGYTTCTTCC-3′. The PCR amplification was performed in 25 µL volume containing 5 ng viral cDNA, 1 × PCR buffer, 0.4 mM dNTPs, 0.4 mM MgCl2, 0.6 μM specific primer pairs, and 1 unit of Taq polymerase (Qiagen GmbH, Hilden, Germany). The thermal cycling parameters for the outer PCR were an initial denaturation at 94 °C for 5 min, followed by 35 cycles of denaturation (95 °C for 30 s), annealing (54 °C for 30 s), and extension (72 °C for 30 s), followed by a final extension at 72 °C for 10 min. The thermal cycling parameters for the inner nested PCR were an initial denaturation at 94 °C for 5 min, followed by 40 cycles of denaturation (95 °C for 30 s), annealing (56 °C for 30 s), and extension (72 °C for 30 s), followed by a final extension at 72 °C for 10 min. A plasmid containing HEV cDNA served as a positive control. Amplicons (306 bp) were visualized on 1.2% agarose gels stained with SYBR Green.

### 2.6. Statistical Analysis

Based on the confidence interval for a proportion of positive results and the sample size, 95% Cis was calculated. The lower bound and upper bound were determined using the Sample Size Calculator online tool for clinical research planning (https://sample-size.net; accessed on 15 August 2023).

The age of 40 years was selected according to the average value in the range of minimum and maximum value (19–59 years). There was no specific age difference between the two genders.

All analyses were performed employing the R software (version 4.0.5). When comparing differences between and among groups, we used Fisher’s exact or Chi-squared tests where appropriate. The level of significance was set at a *p* value of <0.05.

## 3. Results

### 3.1. Baseline Characteristics

All relevant donor demographic data, including gender, age, self-reported ethnicity, and place of residence, were collected and analysed together with the serological and molecular results. Place of residence was used to categorize the communities to which the donors belong. Each donor was considered only once, i.e., only the first blood donation was included in the analysis. All blood donors were serologically tested negative for HIV, HCV, and HBV (HBsAg). None of them had a previous history of receiving blood transfusion. All donors were from the northern region and represented the urban population of Hanoi, predominantly of Kinh ethnicity (94%, *n* = 520). The median age of the cohort was 34 years with an interquartile range (IQR) of 19–59 years. The blood donor population comprised 451 males and 102 females. None of the female blood donors were pregnant at the time of blood donation. The other demographic parameters showed that 97% (*n* = 536) lived in urban areas and consumed either well or tap water.

### 3.2. HEV Seroprevalence

Serological tests revealed that 26.8% (148/553) were positive for anti-HEV IgG and 0.5% (3/553) were positive for anti-HEV IgM (Table 1).

**Figure 1:**
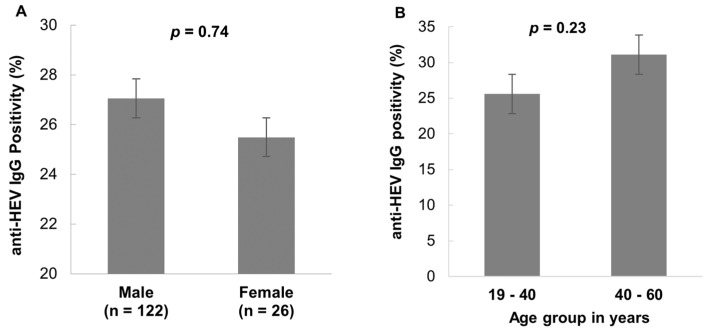
Distribution of seroprevalence of anti-HEV IgG and IgM based on sex (**A**) and age (**B**). Data are provided as percentages. No significant differences were observed between sex (*p* = 0.74) and age (*p* = 0.23).

Male and female donors had high anti-HEV IgG positivity (27.1%, 122/451, and 25.5%, 26/102, respectively), whereas the gender distribution was not statistically significant (*p* = 0.74) (Figure 1A). The age group 40–60 years had a high anti-HEV IgG seroprevalence compared to the age group 19–40 years, but the difference was not statistically significant (*p* = 0.23) (Figure 1B).

**Figure 2:**
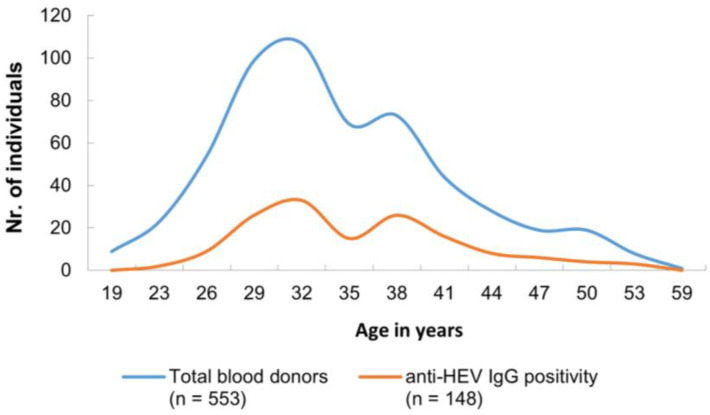
Age-stratified distribution of anti-HEV IgG positivity among all blood donors. The data are provided as absolute numbers. The median age of blood donors was 34 years with an age range of 19–59 years (min.–max.). The majority of anti-HEV IgG-positive cases were between 27 and 40 years old, and this result is a characteristic of the general blood donor population.

Three anti-HEV IgM-positive samples were obtained from three male donors, aged 32, 39, and 42 years, respectively. Observing the age distribution, anti-HEV IgG was detected in individuals as young as 23 years old, while the oldest individual was 54 years old. The majority of anti-HEV IgG-positive cases were between 27 and 40 years old, and this fact is consistent with the characteristics of the general blood donor population (Figure 2).

### 3.3. HEV Nucleic Acid Testing

Nucleic acid testing of conserved regions of the overlapping HEV ORF1 region of a 306 bp fragment revealed no HEV RNA positivity, indicating that there was no evidence of HEV viremia.

## 4. Discussion

Emerging viral infectious diseases are a constant threat in Southeast Asia, including Vietnam, and the safety of blood transfusions could be compromised as the region remains a hotspot for emerging infectious diseases, making epidemiological surveillance for transfusion-transmitted viruses indispensable. While HEV infection is asymptomatic in immunocompetent blood donors, HEV-infected blood transfusion products can exacerbate the clinical course of HEV infection in immunocompromised patients. In this study, we report about HEV exposure in the blood donor population. Although there was no evidence of HEV viremia, we found a high prevalence of HEV IgG seropositivity. HEV is increasingly recognized in Vietnam, and there are no practices of HEV screening, especially in one of the main hospitals where most blood donations are conducted. While European countries such as Germany, Spain, the Netherlands, Iceland, France, and the United Kingdom have introduced mandatory HEV screening of blood donors [2], HEV is not integrated in routine blood donor testing in Vietnam. In Vietnam, testing is only recommended for five transfusion-transmissible infectious agents, namely HIV, HBV, HCV, *Treponema pallidum*, and *Plasmodium* spp.

Previous studies from Vietnam reported an anti-HEV IgG seroprevalence in the general population of 31% [7], which is comparable to the anti-HEV IgG seroprevalence observed in this study (26.8%). A very low anti-HEV IgM seroprevalence (0.5%) was observed in this study compared to other studies in Vietnam in occupationally unexposed individuals (6%) [7] and in pregnant women in the third trimester (2%) [4]. When comparing HEV seroprevalence in individuals who have frequent contact with pigs, such as pork vendors, pig farmers, and slaughterhouses workers, significantly high seropositivity for anti-HEV IgG (53%) and anti-HEV IgM (11.3%) was found in a study in Northern Vietnam [7]. These exposed individuals are undoubtedly at an increased risk of zoonotic HEV transmission. Comparing HEV seroprevalence with European countries showed that the rate of seropositivity varies widely among different countries and regions. In the Netherlands, the seroprevalence for anti-HEV IgG was 27% using the same test systems as in our study [12]. Another study in South–West France found that a high proportion of blood donor were positive for anti-HEV IgG (53%) [17]. In contrast, low seroprevalence of anti-HEV IgG was found in adults in Germany (15%) [18] and among blood donors in Italy (9%) [19]. Although the majority of seropositive cases in our study were male, no significant difference in seroprevalence rates was observed between the sexes (males 27.1% versus females 25.5%) (Figure 1A). In addition, there are no differences in seroprevalence across different age distributions in this study (Figure 1B). Given the geographic differences in seroprevalence in different study cohorts within Vietnam, our data provide valuable information about HEV exposure in a healthy adult and relatively young blood donor population. However, other studies on both white and Asian ethnic groups, and particularly in the male population, showed that hepatitis E risk increases significantly with age [20,21], and accordingly, men are much more likely to develop symptomatic hepatitis E [22].

The incubation period of HEV lasts 2–9 weeks (15–64 days) in the case of acute infection, and RNA can usually be detected in the blood 2–6 weeks after infection, while HEV-specific IgM antibodies appear in the bloodstream 3–4 weeks after infection and persist between four and six months [2]. Our results showed that <1% of blood donors were tested positive for anti-HEV IgM but did not have HEV viraemia, indicating recent exposure and that detection with molecular testing must have resulted in false negatives due to the diagnostic time window. Similar to what was observed in this study, HEV RNA viraemia and/or HEV RNA positivity was low in Europe (France: 0.045%; Denmark and Spain: 0.03%; and Germany: 0.08%) and in Asian countries (China: 0%) [12,14]. In addition, the HEV subgenotypes 3c, 3e, and 3f reported in these studies suggest a zoonotic transmission pattern among blood donors, as these genotypes have been frequently detected in domestic pigs and wild boar in Europe [23,24,25]. This pattern of HEV infection has been increasingly recognized in several European countries, and in Vietnam, where HEV genotype 3 is prevalent [7]. An epidemiological link has been established between hepatitis E cases and consumption of undercooked pork, clearly indicating a zoonotic transmission consistent with our HEV IgG seroprevalence results [7,16].

A limitation of the study is that the blood donors were representative of the healthy adult population living in urban areas in the northern region of Vietnam and were geographically distinct. There may be differences in local exposure risks in other rural areas and highland regions, but further studies are needed to investigate this observation in other communities. Another limitation of our study could be the sensitivity for our RT-PCRs, which are approximately 1 × 10^3^ copies/mL (approx. 840 IU/mL). Thus, it cannot be ruled out that individuals with a lower HEV viral load might have been tested false negative although they were HEV IgM positive. However, a recent study by Lhomme et al. [26] showed that in asymptomatic blood donors, a median HEV load of 717 IU/mL was determined, and symptomatic individuals showed a median load of 2.82 × 10^5^ IU/mL. Therefore, the PCR detection limit was acceptable for this study analysing apparently healthy individuals showing no hepatitis E-like symptoms. Overall, while the absence of HEV viraemia in blood donors from Northern Vietnam is encouraging, further surveillance studies will help unravel the burden of the disease in the sub-region.

## 5. Conclusions

The HEV IgG seroprevalence in the blood donor population studied in Northern Vietnam is high (26.8%), although the proportion of HEV IgM antibodies is low (<1%), and no HEV RNA was detected. Therefore, according to the results of the present study, the risk of transmission of HEV infection from Vietnamese blood donors to, for example, immunocompromised patients, is considered to be rather low. Nevertheless, from a public health policy perspective, HEV screening of blood samples, as regulated in few European countries, is recommended to minimize the risk of chronic hepatitis E associated with acute liver inflammation, especially in immunocompromised patients. Further epidemiological surveillance is warranted to confirm our data in other geographical areas of Vietnam to rule out transfusion-transmitted HEV.

## Figures and Tables

**Table 1:** Hepatitis E virus IgG and IgM seroprevalences in investigated blood donors.

Biomarkers	*n* (%)	95% CI
anti HEV-IgG	148/553 (26.8)	23.1–30.6
anti HEV-IgM	3/553 (0.5)	0.1–1.5

## Data Availability

All data generated or analysed during this study are included in this article.
